# Nephropathogenic infectious bronchitis virus induces epithelial-mesenchymal transition of renal tubular epithelial cells through the TGF-β/p-P38 pathway causing uric acid excretion disorder in chickens

**DOI:** 10.1128/jvi.01031-25

**Published:** 2025-10-14

**Authors:** Yunfeng Chen, Yan Shi, Cheng Huang, Haoyu Huang, Yizhou Zeng, Gaofeng Cai, Zhanhong Zheng, Ping Liu, Xiaona Gao, Xiaoquan Guo

**Affiliations:** 1 Jiangxi Provincial Key Laboratory for Animal Health, College of Animal Science and Technology, Jiangxi Agricultural University 91595, Nanchang, Jiangxi, China; 2 School of Computer and Information Engineering, Jiangxi Agricultural University 91595, Nanchang, Jiangxi, China; University of North Carolina at Chapel Hill, Chapel Hill, North Carolina, USA

**Keywords:** NIBV, uric acid, ABCG2, EMT, TGF-β/p-p38 pathway

## Abstract

Nephropathogenic infectious bronchitis virus (NIBV) infection usually causes kidney enlargement and urate deposition in chickens, leading to sudden death. However, the mechanism by which NIBV causes urate deposition in the kidneys has not yet been elucidated. The coordinated operation of uric acid transporters is crucial for the kidneys to maintain uric acid homeostasis, and existing studies have shown that the occurrence of cell epithelial–mesenchymal transition (EMT) can affect the expression of uric acid transporters. Thus, this study aimed to explore the effect of NIBV on urate transporters and elucidate the mechanism of EMT in NIBV-induced urate deposition in chicken kidneys *in vivo* and *in vitro*. The results revealed that NIBV infection led to an abnormal increase in uric acid levels in chicks, affected the expression of uric acid transport proteins, induced EMT in renal tubular epithelial cells, and activated the TGF-β/p-p38 pathway. The changes in uric acid concentration induced by NIBV were related to the uric acid excretion protein ABCG2, whose expression is negatively regulated by EMT. The occurrence of NIBV-induced EMT coincided with the time point at which NIBV activated the TGF-β/p-p38 pathway. After siRNA knockdown of p38 MAPK, EMT did not occur, and ABCG2 expression returned to normal. In summary, the mechanism by which NIBV causes abnormal elevation of uric acid levels in chickens is through the induction of EMT in renal tubular epithelial cells via the TGF-β/p-p38 pathway, which strongly inhibits the expression of ABCG2, thereby causing uric acid excretion disorders in chickens.

**IMPORTANCE:**

NIBV infection results in a reduction in uric acid transporter expression in the kidneys of chickens. ABCG2 plays a pivotal role in the excretion of uric acid in chickens. The mechanism by which NIBV causes an abnormal increase in uric acid levels in chickens involves the induction of renal tubular epithelial cell EMT through the TGF-β/P-p38 pathway and the subsequent strong inhibition of ABCG2 expression, causing uric acid excretion disorders in chickens.

## INTRODUCTION

Poultry serves as the primary source of nutritional protein for human consumption (1). However, the widespread prevalence of infectious bronchitis virus (IBV) within the poultry industry has significantly compromised poultry health (2, 3). Chickens of all ages are susceptible to IBV infection, with younger chickens exhibiting more severe clinical manifestations than their older counterparts (4–6). Nephropathogenic infectious bronchitis virus (NIBV) is a branch of IBV, with its primary target organ being the kidneys. NIBV occurs mainly in Asian and Middle Eastern countries and poses a major threat to the chicken industry (7). Our previous research demonstrated that NIBV infection results in kidney enlargement, urate deposition, and the occurrence of white loose stools in poultry. These conditions not only increase mortality rates among affected poultry but also impose significant economic burdens on the poultry breeding sector (8, 9). Urate deposition is typically linked to elevated levels of uric acid (UA), as prolonged high concentrations of uric acid lead to the precipitation of urate in the bloodstream and its subsequent accumulation in the internal organs of poultry (8). Therefore, investigating the mechanisms through which NIBV induces an abnormal increase in UA levels in chicks is highly important.

UA is generated through purine metabolism. Under normal conditions, the production and excretion of uric acid in poultry maintain a dynamic equilibrium (9). When the body consumes an excessive amount of purine-rich foods or when the kidneys encounter disorders in uric acid excretion, this balance is disrupted (8). The kidney accounts for up to two-thirds of the excretion of urate and constitutes an essential organ for regulating uric acid homeostasis (10). The regulation of uric acid homeostasis by the kidney depends on uric acid transporters located in renal tubular epithelial cells. Uric acid transporters are categorized into uric acid reabsorption proteins and uric acid excretion proteins, which play roles in reabsorbing and secreting uric acid, respectively (11, 12). Currently, uric acid transporters have been extensively investigated; renal uric acid transporters mainly belong to the organic anion transporter (OAT) family, the glucose transporter glucose transporter (Slc2a9) family, the ATP-binding cassette transporter superfamily (ABCs), the sodium phosphate cotransporter (NTP) family, and others (13). ABCG2 is an ATP-dependent urate export pump that plays a crucial role in the transport of uric acid in cells, significantly contributing to uric acid excretion within the body (14). However, whether uric acid transporters are involved in NIBV-induced gout in chickens has not yet been reported.

Epithelial‒mesenchymal transition (EMT) is a biological process in which epithelial cells transform into cells with a mesenchymal phenotype, and this process constitutes the early basis of fibrosis (15). During this process, alterations can occur in both the phenotypes of epithelial cells and gene expression profiles, manifested by diminished cell adhesion, enhanced migratory ability, and changes in cell morphology (16). During EMT, the expression of the epithelial cell marker E-cadherin is downregulated (17), and the expression of the fibroblast marker fibronectin (FN) is significantly decreased (18). Although multiple signals can regulate EMT, transforming growth factor-β (TGF-β) typically plays a predominant role as an inducer of EMT (19–21). Additionally, studies have demonstrated that TGF-β-induced EMT potently inhibits ABCG2 expression and that the removal of TGF-β can restore the cell phenotype to that of an epithelial type and reinstate ABCG2 expression (22). Hence, TGF-β-induced EMT is associated with the uric acid excretion-related protein ABCG2. Furthermore, studies have confirmed that the p38 MAPK signaling pathway is closely linked to MET (23, 24). p38 MAPK serves as a downstream gene of TGF-β and is capable of being activated by the latter. The regulation of signal transduction and activation within this pathway is governed by the expression level of TGF-β. When TGF-β binds to receptors on cells, it can phosphorylate MKK4, and P-MKK4 can phosphorylate p38 to p-p38, which subsequently enhances the promoters of genes related to EMT (15, 25).

Research has indicated that coronaviruses are closely associated with EMT and that infection by coronaviruses can lead to an increase in fibrin levels within the organism, resulting in tissue fibrosis (26, 27). For example, the new severe acute respiratory syndrome coronavirus 2 (SARS-CoV-2) virus that emerged in 2019 can induce pulmonary fibrosis (PF) in infected individuals, and its mechanism includes promoting the expression of TGF-β (28). In addition, Reyhaneh Niayesh-Mehr et al. elucidated EMT-related processes related to the origin of PF in COVID-19 through the latest investigation of cellular and molecular mechanisms (29). Coincidentally, NIBV is classified into the family Coronaviridae and the genus Gamma coronavirus*.* Therefore, we hypothesized that EMT may occur in the kidney tissue of chicks infected with NIBV.

This study aims to elucidate the mechanism through which NIBV induces abnormal elevations in serum uric acid levels in the body by exploring the impact and mechanism of NIBV infection on kidney uric acid transporters in chicks. Our research elucidated the mechanism through which NIBV infection leads to urate deposition in chicks, thereby providing a theoretical foundation for the prevention and treatment of avian gout.

## MATERIALS AND METHODS

### Animal feeding and sample collection

A total of 300 one-day-old healthy Hy-Line brown chickens were raised in the laboratory of the College of Animal Science and Technology at Jiangxi Agricultural University with free access to drinking water and food. At 28 days of age, the chicks were randomly divided into a control group (Con) consisting of 100 chicks and a model group (NIBV) of 200 chicks. The model group was inoculated with the SX9 virus (10^5 ELD_50_/0.2 mL), while the control group was inoculated with physiological saline. To prevent cross-infection, contact between the two groups of chickens was avoided. Kidney tissues were collected at 1, 3, 5, 7, 9, 11, 13, 15, 18, 21, and 28 days post-infection (dpi) and stored at −80°C until they were used in subsequent experiments. The isolated IBV SX9 strain was deposited at the College of Animal Science and Technology of Jiangxi Agricultural University, and its specific sequence was identified as MN707951.1 in the NCBI database.

### Chicken renal tubular epithelial cell isolation, culture, and treatment

In accordance with previous methods (30), primary renal tubular epithelial cells were extracted from 1- to 7-day-old chicks for *in vitro* experiments. The isolated chicken primary renal tubular epithelial cell suspension was cultured in low-sugar DMEM (Solarbio, Beijing, China) supplemented with 10% FBS (Excel, Shanghai, China), adjusted to a seeding density of 1 × 10^6 cells/mL, and cultured in a cell incubator at 37°C with 5% CO_2_. After 24 h of incubation, the original culture medium was discarded, and the cells were washed once with PBS (Sevier, Wuhan, China). The cells were subsequently cultured in DMEM supplemented with 5% FBS until they reached confluence (70–80%) within the culture dish.

When the cell density reached approximately 70–80%, the cells were processed. The control group (Con, C) was treated with pure DMEM (Solarbio, Beijing, China) for 2 h, and the NIBV infection group (NIBV, N) was infected with 1 MOL of virus based on TCID50. During this two-hour treatment period, the cell culture plate was gently shaken every half hour. Afterward, the medium was switched to DMEM containing 2% FBS and 1% double antibiotics for continued culture.

The specific small interfering RNA (siRNA) targeting p38 (sip38) used in this study was purchased from HANBIO (Shanghai, China). In accordance with the manufacturer’s instructions, siRNA was transfected using RNA Fit Transfection Reagent (HANBIO, HH20250220WY-SI02) at a final concentration of 20 nM. CY3 siRNA NC (sense strand: UUCUCCGAACGUGUCACGUTT; antisense strand: ACGUGACACGUUCGGAGAATT); siRNA NC (sense strand: UUCUCCGAACGUGUCACGUTT; antisense strand: ACGUGACACGUUCGGAGAATT); Si-p38MAPK3 (sense strand: CGAUGAAGUAAUCAGCUUUGUTT; antisense strand: ACAAAGCUGAUUACUUCAUCGTT).

### Cell viability assay

Cell viability was determined using CCK-8 detection reagent according to the manufacturer’s instructions (YEASEN, Shanghai, China). Renal tubular epithelial cells were spread in a 96-well plate. After adhesion and growth, the cells were cultured with different concentrations of inhibitors (0, 2, 4, 6, 8, 10, 12, and 14 µg/mL) for 24 h. After the cells were washed once with PBS, 110 µL of CCK-8 reagent was added to each well, and the cells were incubated at 37°C for 2 h in the dark. Afterward, the absorbance value (OD) of each well was measured at 450 nm using a microplate reader, and the cell viability was calculated as the OD. Each concentration was established in eight replicate wells.

### Measurement of serum uric acid content

The collected serum was used to measure the UA concentration in the serum using a fully automatic biochemical detection analyzer (Hitachi Group, 3100, Japan).

### Total RNA extraction and real-time RT‒PCR

RNA was extracted from kidney tissue and cell samples using the TRIzol method (Vazyme, Nanjing, China). The RNA concentration was measured and adjusted to 1000 ng before it was reverse transcribed into cDNA. The reverse transcription system and PCR amplification were performed following the instructions of the reverse transcription and qPCR kit, and the amplification procedure was 94 predenaturation at 94°C for 5 min and denaturation at 94°C for 50 s, annealing at 56°C for 30 s, and extension at 72°C for 1 min for 38 cycles. The fluorescence quantitative PCR system was prepared according to the instructions of the fluorescence quantitative kit (TransGen, Beijing, China). The primers were designed on NCBI and synthesized by Shanghai Qingke Biotechnology Co., Ltd. The gene sequences are shown in Table 1.

**TABLE 1:** Gene primer sequences

Gene name	Accession number	Primer sequence (5′−3′)
GAPDH	NM_204305.1	F: TGGCATCCAAGGAGTGAGC
		R: GGGGAGACAGAAGGGAACAG
ABCG2	NC_052537.1	F: CAGCAAGCAAGGAAGATCAC
		R: GGCTGGAGTTGAGATACTTC
ABCC4	NM_001030819	F: TAGTGTTGGTCAGAGACAGC
		R: GTGCAATGGTCAGAACTGTG
SLC2A9	XM_046940740.1	F: ACCAGCGTGGAAGGTACTTG
		R: AGATGACCCAAAAGCACCAGT
E-cadherin	NM_001039258.3	F: GACAGGGACATGAGGCAGAA
		R: GCCGTGACAATGCCATTCTC
FN	NM_001198712.2	F: GGCGAGGAGTGGGAGAGATT
		R: CGTGGCACCACTTAGAGGAA

### Protein extraction and western blot

Kidney tissue and cell samples were digested and lysed using a preprepared lysis solution of RIPA: phosphatase inhibitor: protease inhibitor = 100:1:1 (Solarbio, Beijing, China). After lysis, the supernatant was centrifuged, and the concentration of the supernatant was determined with a BCA kit (Solarbio, Beijing, China), which was set to 5 mg/mL (tissue) or 1.5 mg/mL (cell). After loading buffer was added to the sample, it was placed in a water bath at 100°C for 10 min to denature the protein. In this way, the protein sample was prepared before storage at −80°C for later use.

The WB process involved electrophoresis at 80–120 V, wet transfer at 300 mA, and blocking with Fast Blocking Solution (Epizyme, Shanghai, China) for 20 min. The membranes were washed with PBST three times for 10 min, incubated with primary antibody at 4°C overnight, and washed again with PBST three times for 10 min. The sections were incubated with a goat secondary antibody for 40 min and finally developed using ECL supersensitive luminescent solution (Abbkine, Wuhan, China). The protein bands were quantified using Image J, and the resulting protein bands were recorded and saved for statistical analysis. The primary antibodies used for WB were ABCG2, FN, MKK4, P-MKK4, TGF-β (Wanleibio, Shenyang, China), ABCC4 (ABclonal, Wuhan, China), SLC2A9, E-cadherin, p-p38, p38 (Abmart, Shanghai, China), and the secondary antibody (Proteintech Group, Wuhan, China).

### Determination of viral load

The pEASY-T3-N–positive plasmid was constructed according to the laboratory’s previous method (31), the number of virus copies in the positive plasmid was calculated, and a standard curve was established by equal dilution. The absolute fluorescence quantitative PCR method was used to measure the viral load in the kidneys of the chicks at each time point.

### Immunofluorescence staining

During sampling at 1, 5, 11, and 18 dpi, portions of the kidney tissue were excised and preserved in 4% paraformaldehyde for subsequent embedding and the preparation of wax blocks. The cell slides were fixed with 4% paraformaldehyde following NIBV treatment for 6, 12, 18, and 24 h. After dewaxing, antigen retrieval, and serum blocking, the wax blocks were incubated overnight at 4°C with the appropriate primary antibody. The corresponding secondary antibody was subsequently added, and the samples were incubated at 37°C for 1 h. Finally, after the nuclei were stained with DAPI, anti-fade mounting medium was added to the tissue, and a coverslip was placed on top. The prepared fluorescent slides were stored at 4°C in the dark for later observation via fluorescence microscopy.

### Cell scratch test

When the cell density reached 70% to 80%, viral infection treatment was performed, and uniform scratches were made on the bottom of the culture dish. Observation and imaging under the microscope were carried out at 6, 12, 18, and 24 h post-scratching, with three replicate wells for each group, and the experiment was repeated three times.

### Statistics

In this experiment, Image J was used to conduct grayscale analysis of protein bands and quantify the scratch area. All the data were statistically analyzed using GraphPad Prism 9.0 software. The independent sample *t*-test was used to compare the differences between the two groups. One-way analysis of variance (ANOVA) was used to compare the differences between multiple groups. The data are displayed as the means ± SD. The data were graphed using GraphPad Prism 9.0 software. The threshold for statistical significance was *P* < 0.05 (*), *P* < 0.01 (**), and *P* < 0.001 (***); if *P <* 0.05, the difference was considered significant.

## RESULTS

### Determination of pathological modeling results and time period *in vivo*

**Fig 1:**
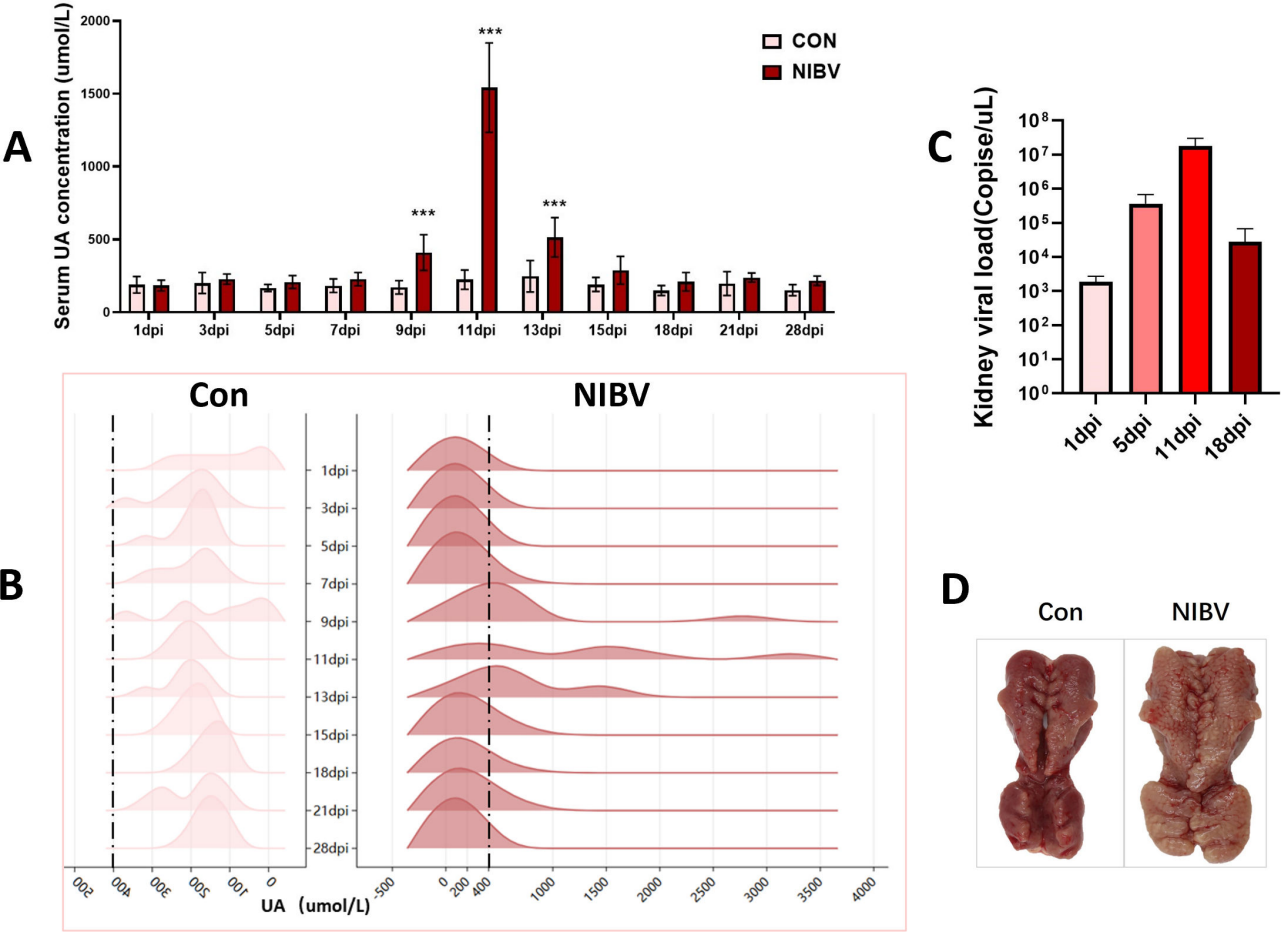
NIBV modeling scenario *in vivo*. (**A**) Serum uric acid (UA) concentration measured at 1, 3, 5, 7, 9, 11, 13, 15, 18, 21, and 28 dpi. (**B**) The peak and peak diagram reflects the distribution of uric acid concentration in Con and NIBV groups in each time period. (drawn on the Chiplot website: https://www.chiplot.online/) (*n* = 8). (**C**) qPCR detects renal viral load at 1, 5, 11, and 18 dpi. The values were expressed in the format mean ± SD (*n* = 6). (**D**) Kidney anatomy observed at day 11 dpi. Normal group (control) and model group (NIBV). The values were expressed in the format mean ± SD (*n* = 6). ***P < 0.001 compared with CON.

Analysis of serum uric acid concentration revealed that uric acid levels in the model group (NIBV) began to increase at 5 dpi compared with control group (Con). This increase reached its peak at 11 dpi, followed by a gradual decline, and eventually returned to baseline levels by 18 dpi (Fig. 1A). The uric acid concentration distribution diagram revealed that the UA concentration in the Con group was less than 400 µmol/L at all time points, whereas at 9, 11, and 13 dpi, 50% of the chicks in the NIBV group had a UA concentration exceeding 400 µmol/L, and at 11 dpi, it was even greater than 2000 µmol/L (Fig. 1B). Concurrently, the renal viral load also reached its maximum at 11 dpi before gradually decreasing thereafter (Fig. 1C). Renal anatomy at 11 dpi revealed that the kidneys in the NIBV group were significantly enlarged and had urate deposition (Fig. 1D).

### Expression results of genes and proteins related to uric acid transport

**Fig 2:**
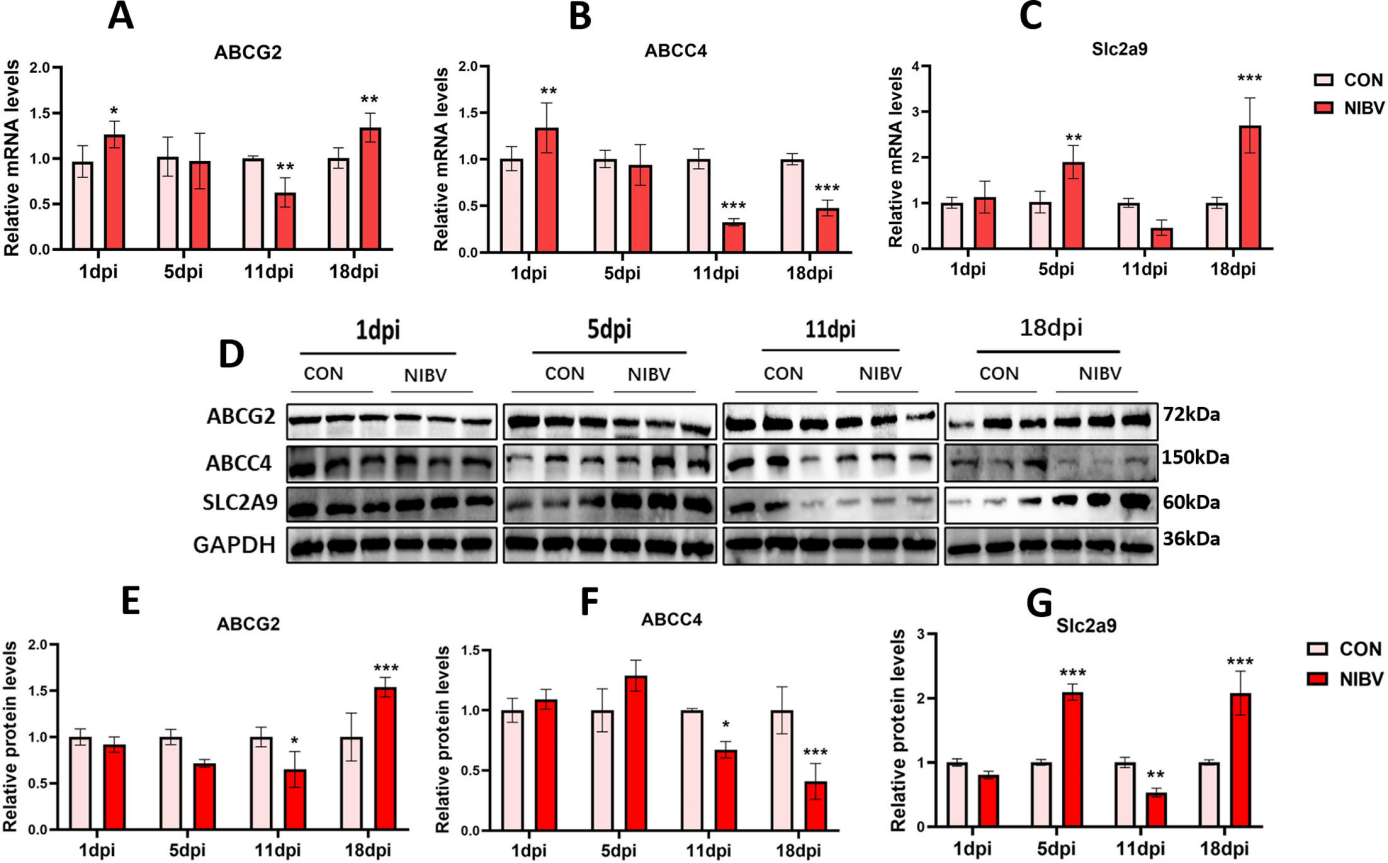
Gene and protein expression of uric acid transport-related molecules at 1, 5, 11, and 18 dpi. (**A**) Results of qPCR detection for ABCG29 (**A**), ABCC4 (**B**), and Slc2a9 (**C**) are presented (*n* = 6). (**B**) Western blot bands for ABCG2, ABCC4, and Slc2a9 are shown. (**D**) Quantitative analysis results of the gray values for ABCG29 (**E**), ABCC4 (**F**), and Slc2a9 (**G**) are provided. The densitometric values of the various proteins were normalized to GAPDH protein levels (*n* = 3). Values are expressed as mean ± SD. *P < 0.05, **P < 0.01, ***P < 0.001 compared with the control group (CON).

Based on the previously measured viral load and serum uric acid concentration results, we selected four time periods—1, 5, 11, and 18 dpi—for subsequent experiments. To explore the influence of NIBV infection on kidney uric acid transport-related proteins in chicks, we detected the molecular expression of the reabsorption-related protein Slc2a9 and the uric acid excretion proteins ABCG2 and ABCC4 through qPCR and WB (Fig. 2A through F). Compared with that in the Con group, the expression of these transporters in the NIBV group decreased significantly at 11 dpi. Interestingly, at 18 dpi, compared with that in the Con group, the expression of ABCC4 in the NIBV group still tended to significantly decrease, whereas the expression of ABCG2 tended to significantly increase.

### Localization and expression of uric acid excreting protein in the kidney

**Fig 3:**
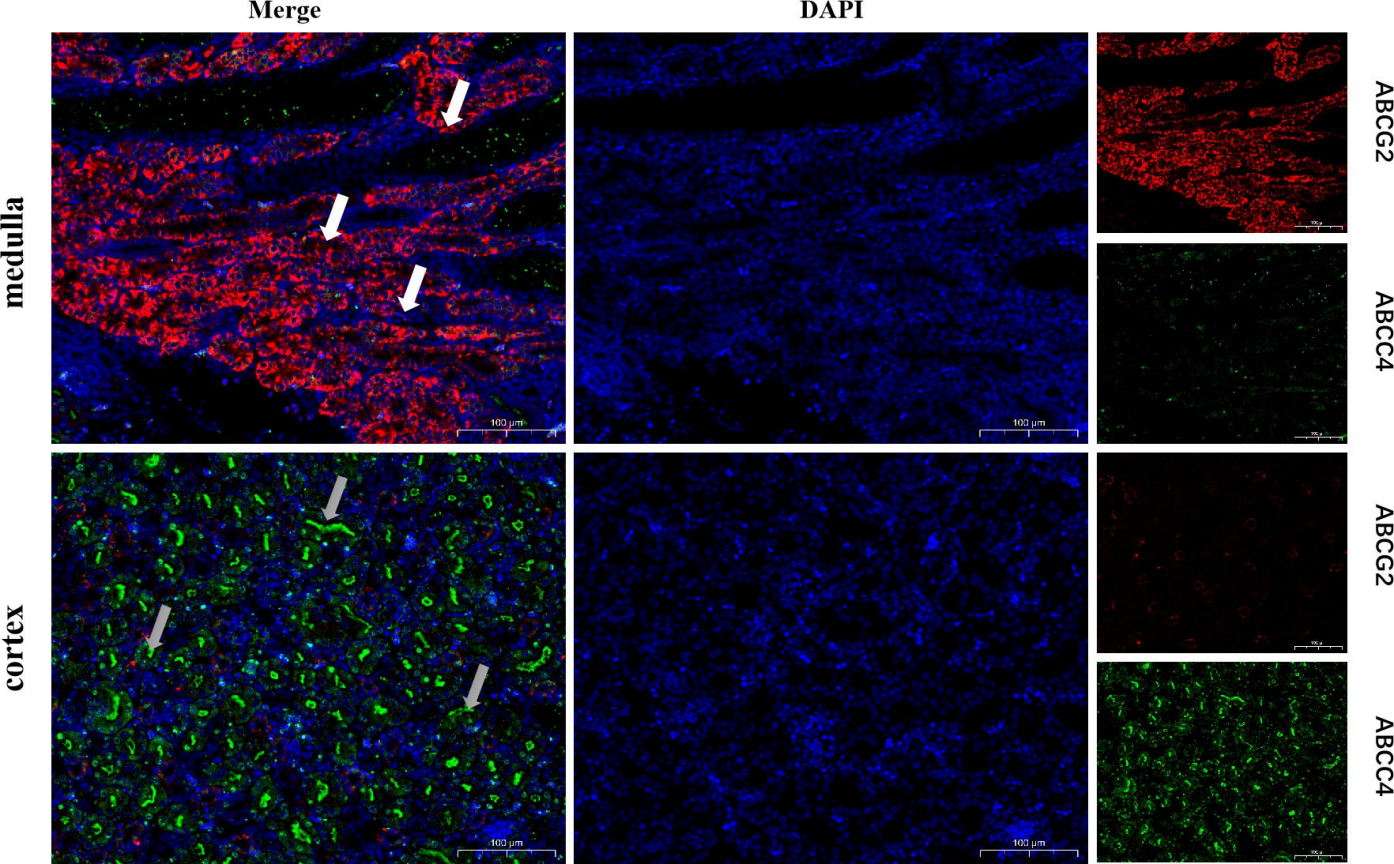
Localization and expression of ABCG2 and ABCC4 in normal chicken kidney. medulla, medulla region of the kidney. cortex, cortical area of the kidney. The red fluorescent marker is ABCG2, and the green fluorescent marker is ABCC4. DAIP nuclei stain blue.

To elucidate why the expression trends of ABCG2 and ABCC4 in the NIBV group were completely opposite at 18 dpi, we selected the kidney tissue of healthy chicks for immunofluorescence costaining of ABCC4 and ABCG2. The results indicated that ABCG2 was expressed mainly in the medullary area of chicken kidneys, whereas ABCC4 was expressed in the cortical area. Additionally, ABCG2 (green fluorescence) was expressed mainly in the kidney on the luminal side of the tubule, and ABCC4 (red fluorescence) was expressed mainly in the basement membrane and apical mold of renal tubular epithelial cells (Fig. 3). In general, those expressed on the basement membrane are mainly responsible for the reabsorption of uric acid, whereas those expressed on the apical membrane are responsible for the excretion of uric acid (32, 33). Combined with the previous data, the uric acid level returned to normal at 18 dpi, and simultaneously, the expression of ABCG2 also returned to normal, while the expression of ABCC4 was irreversibly impaired. We speculate that ABCG2 is a major factor in the excretion of uric acid in poultry.

### Expression of genes related to mesenchymal transformation and pathway-related genes and proteins

**Fig 4:**
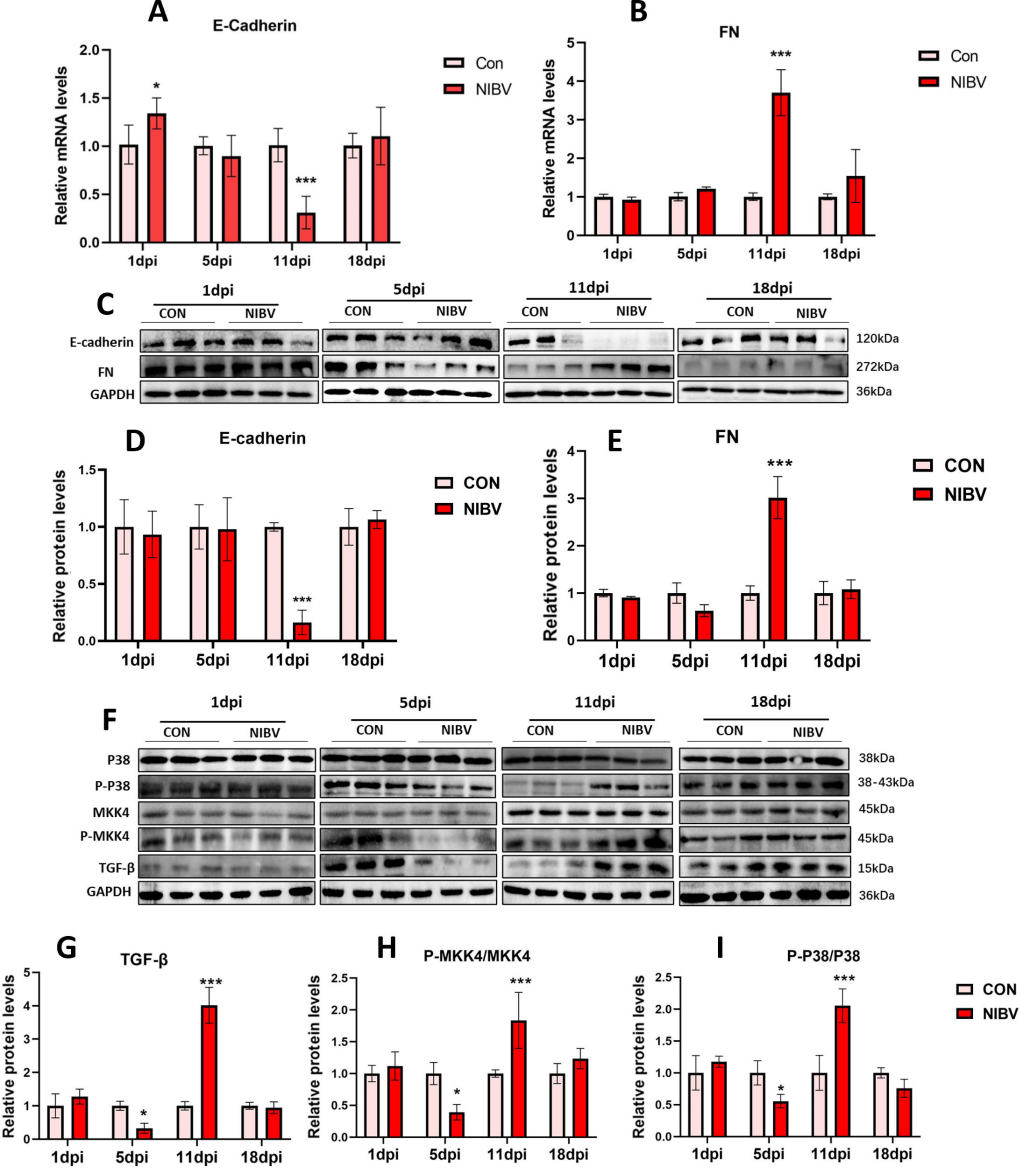
Transcription and translation levels of proteins related to the EMT and the p38 MAPK pathway. (**A and B**) qPCR analysis of EMT-related genes: E-cadherin (**A**) and fibronectin (FN) (**B**) (*n* = 6). (**C**) Western blot bands for EMT-related proteins. (**D and E**) Quantitative gray value analysis of EMT-related protein bands: E-cadherin (**D**) and FN (**E**) (*n* = 3). (**F**) Protein bands associated with the p38 MAPK pathway. (**G, H, I**) Quantitative gray value analysis of protein bands related to the p38 MAPK pathway. The densitometry values of various proteins were normalized to GAPDH protein levels (*n* = 3). The values were expressed in the format mean ± SD. *P < 0.05, **P < 0.01, ***P < 0.001 compared with CON.

Studies have demonstrated that the expression of ABCG2 is inhibited by TGF-β-induced EMT (22). To explore whether ABCG2, the main contributor to uric acid excretion, is affected by EMT, we detected the expression of E-cadherin and FN. The results indicated that compared with that in the Con group, the expression of FN significantly increased and that of E-cadherin significantly decreased in the NIBV group at 11 dpi (Fig. 4A through E), suggesting that EMT occurred in the kidney at this time. During the period when EMT occurs, the expression of ABCG2 is strongly inhibited.

Moreover, studies have shown that p38-MAPK signaling is among the pathways that mediate EM (23, 24). We explored whether NIBV-induced EMT is mediated through the p38-MAPK pathway. Compared with those in the control group, the levels of TGF-β in the NIBV group significantly increased at 11 dpi (Fig. 4G), and the phosphorylation levels of MKK4 and p38 MAPK significantly increased (Fig. 4H and I), indicating that the TGF-β/p-p38 pathway was significantly activated at 11 dpi and that its activation time coincided with the occurrence of EMT.

### Transcriptional level of extracellular uric acid transport proteins and the impact of NIBV infection on renal tubular epithelial cells

**Fig 5:**
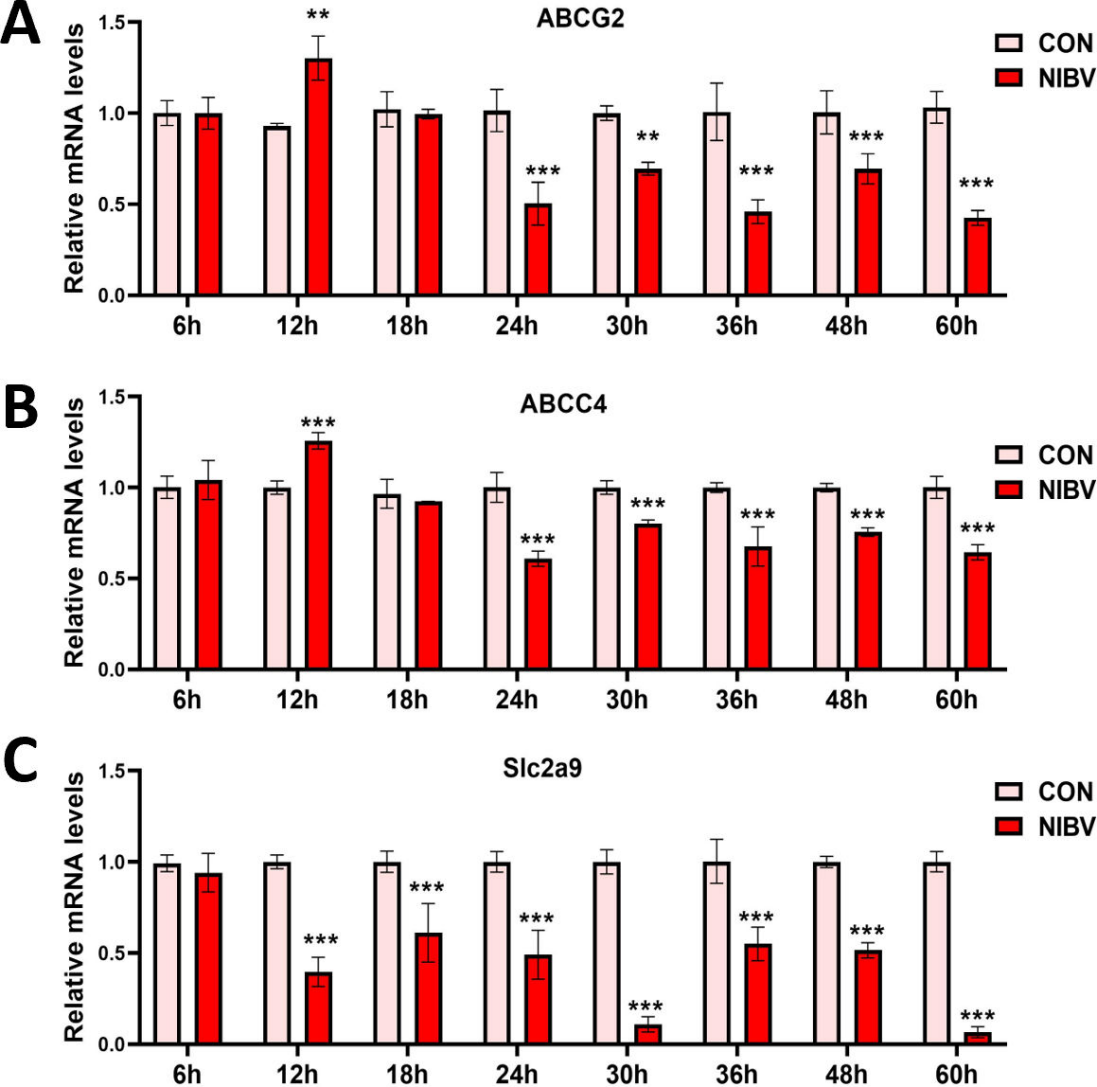
Gene expression of uric acid transport-related proteins *in vitro* at different time periods. ABCG2 (**A**), ABCC4 (**B**), and Slc2a9 (**C**). The values were expressed in the format mean ± SD (*n* = 4). **P < 0.01, ***P < 0.001 compared with CON.

To validate the *in vivo* results, we established an *in vitro* model of renal tubular epithelial cells. Based on previous studies, 6 h after the cells were subjected to NIBV treatment, the virus started to enter the cells (34). Hence, cell samples were collected at 6, 12, 18, 24, 30, 36, 48, and 60 h, and qPCR was performed to measure the transcription levels of uric acid transport-related proteins. Compared with that in the control group, the expression of uric acid excretion proteins in the NIBV group significantly increased at 12 h but then significantly decreased after 24 h (Fig. 5A and B). Uric acid reabsorption significantly decreased compared with that in the control group after 12 h (Fig. 5C). Based on these outcomes, we selected four time periods—6, 12, 18, and 24 h—for subsequent experiments.

**Fig 6:**
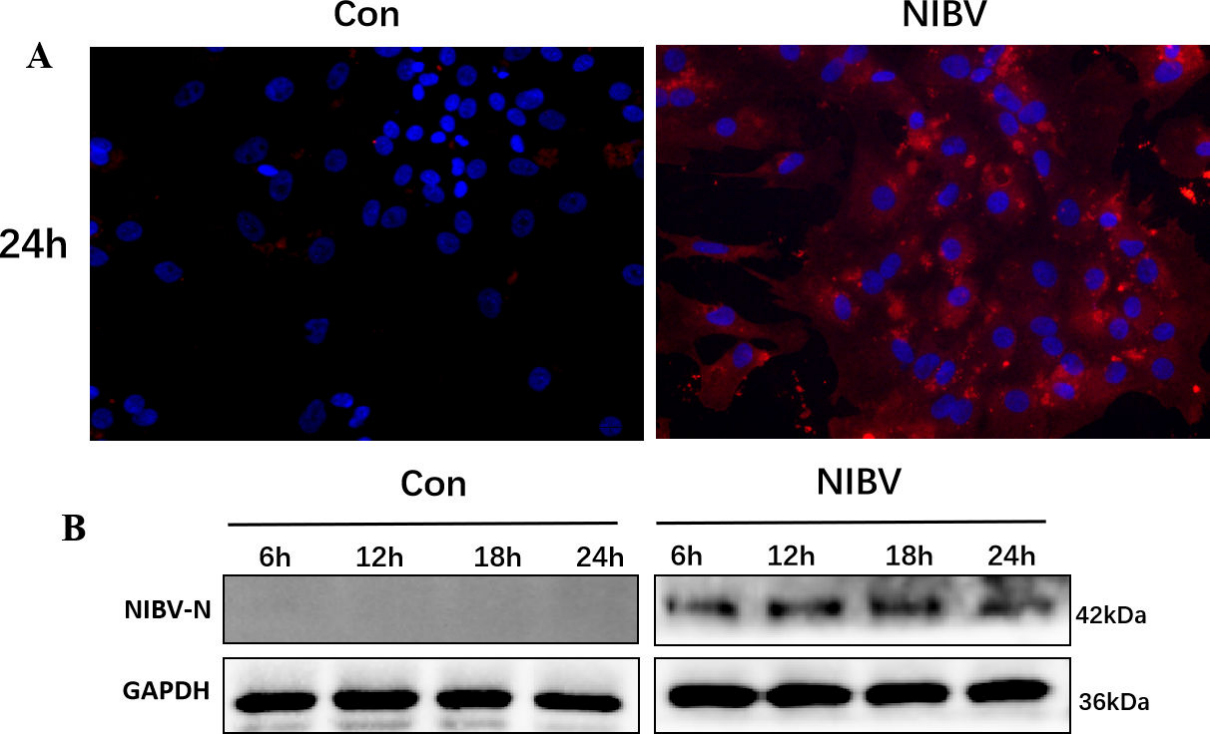
Infection of renal tubular epithelial cells by NIBV. (**A**) Immunofluorescence of NIBV-N protein. The red mark is NIBV-N, and the DAPI stain is blue. (**B**) WB band of NIBV-N protein.

Additionally, by detecting the NIBV-N protein, we demonstrated that NIBV successfully infected primary chicken renal tubular epithelial cells. Immunofluorescence staining revealed that the Con group had almost no red fluorescent spots, whereas the NIBV group exhibited numerous red fluorescent spots (Fig. 6A). WB results indicated that the NIBV-N protein could be detected in renal tubular epithelial cells at 6, 12, 18, and 24 h post-infection (Fig. 6B). These findings suggest that NIBV caused infection in primary chicken renal tubular epithelial cells.

### Expression of uric acid excretion protein *in vitro*

**Fig 7:**
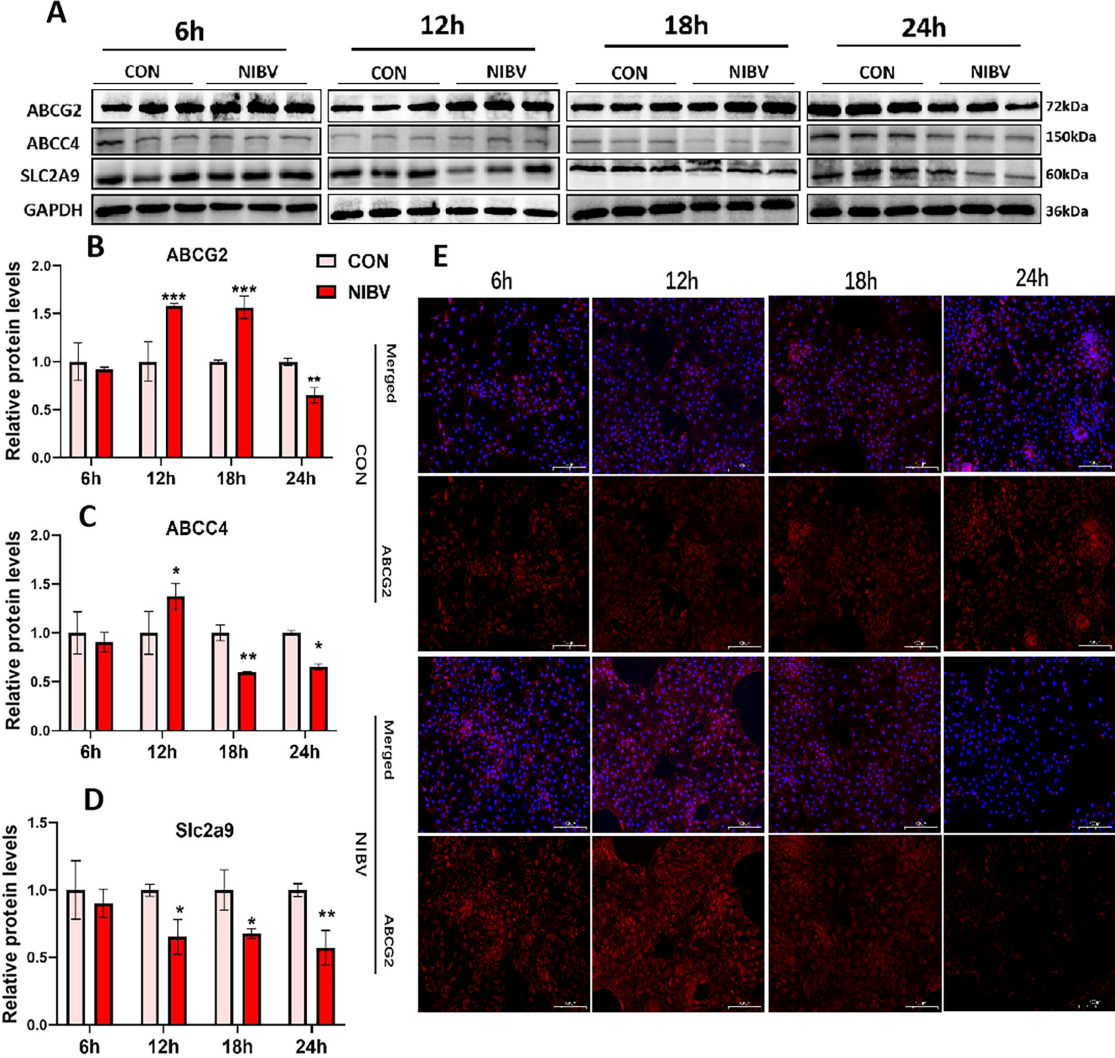
Translation of uric acid transport-related proteins *in vitro* and the localization and expression of ABCG2 in renal tubular epithelial cells. (**A**) WB band of uric acid transport-related proteins. Grayscale quantitative analysis of proteins: ABCG2 (**B**), ABCC4 (**C**), and Slc2a9 (**D**). The densitometry values of various proteins were normalized to GAPDH protein levels (*n* = 3). (**E**) Immunofluorescence of ABCG2. The red mark is ABCG2, and the DAPI stain is blue. The values were expressed in the format mean ± SD (*n* = 3). *P < 0.05, **P < 0.01, ***P < 0.001 compared with CON.

To validate the gene expression levels, we measured the protein levels of uric acid transporters during four time periods. The results demonstrated that the protein expression was in line with the gene expression (Fig. 7A through D). Compared with that in the control group, the fluorescence intensity in the NIBV group increased at 12 h but decreased at 24 h (Fig. 7E).

### Mesenchymal transformation- and pathway-related gene and protein expression results *in vitro*

**Fig 8:**
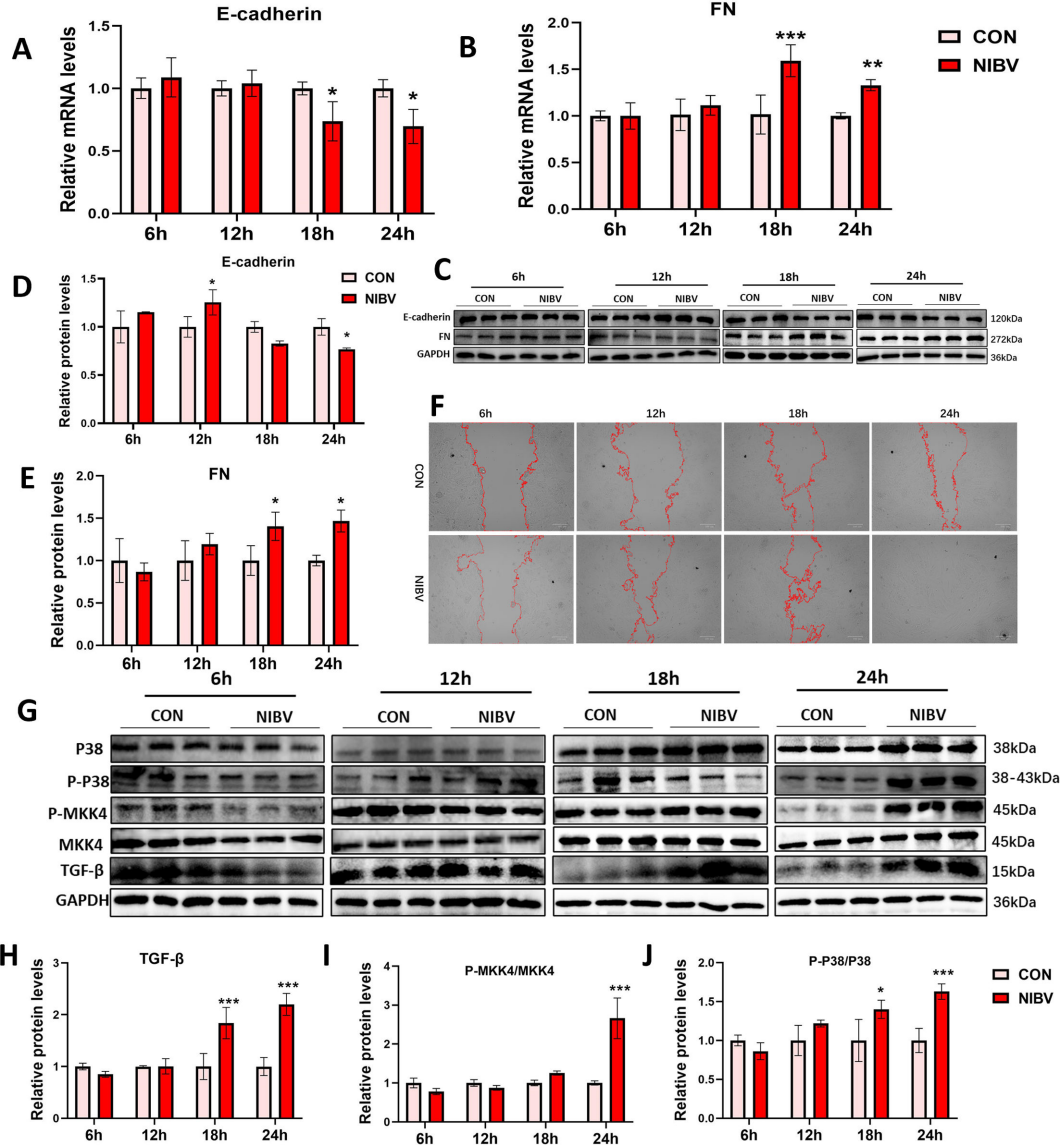
Gene and protein expression of EMT- and TGF-β/p38 MAPK pathway-related genes and proteins at 6 h, 12 h, 18 h, and 24 h *in vivo*. qPCR results of EMT-related genes: FN (**A**) and E-cadherin (**B**) (*n* = 4). (**C**) Protein band of E-cadherin and FN. Grayscale quantitative analysis of E-cadherin (**D**) and FN (**E**) protein bands. (**F**) Scratch test. The cell-free area is between the two red marking lines, and the marked area is identified and marked by ImageJ software. (**G**) WB bands of p38-MAPK pathway-related proteins. Grayscale quantitative analysis of protein. TGF-β (**H**), P-MKK4/MKK4 (**I**), and p-p38/P38 (**J**). The densitometry values of various proteins were normalized to GAPDH protein levels (*n* = 3). The values were expressed in the format mean ± SD (*n* = 3). *P < 0.05, **P < 0.01, ***P < 0.001 compared with CON.

Similarly, we detected the expression levels of EMT and TGF-β/p-38 pathway-related proteins *in vitro.* Compared with that in the control group, the expression of the renal tubular epithelial cell marker E-cadherin significantly decreased at 18 and 24 h, and the expression of the fibroblast marker FN significantly increased in the NIBV group (Fig. 8A through E). Additionally, the results of the scratch assay revealed that the wound healing rate in the NIBV group was faster than that in the control group, especially in the NIBV group, where the scratches were almost completely covered by cells at 24 h, indicating enhanced cell migration ability after NIBV treatment (Fig. 8F). These results suggest that EMT occurred in cells after 18 h of NIBV treatment *in vitro*. The qPCR and WB results demonstrated that, compared with those in the control group, the levels of TGF-β in the NIBV group significantly increased at 18 and 24 h, and the phosphorylation levels of MKK4 and p38 MAPK significantly increased (Fig. 8G through J), indicating that the TGF-β/p-38 pathway was activated at 18 and 24 h and that its activation time coincided with the occurrence time node of EMT. These findings are consistent with the *in vivo* results, suggesting that the p38 MAPK Pathway is likely involved in the initiation and progression of EMT.

### Effect of p38 MAPK knockdown on renal tubular epithelial cells

**Fig 9:**
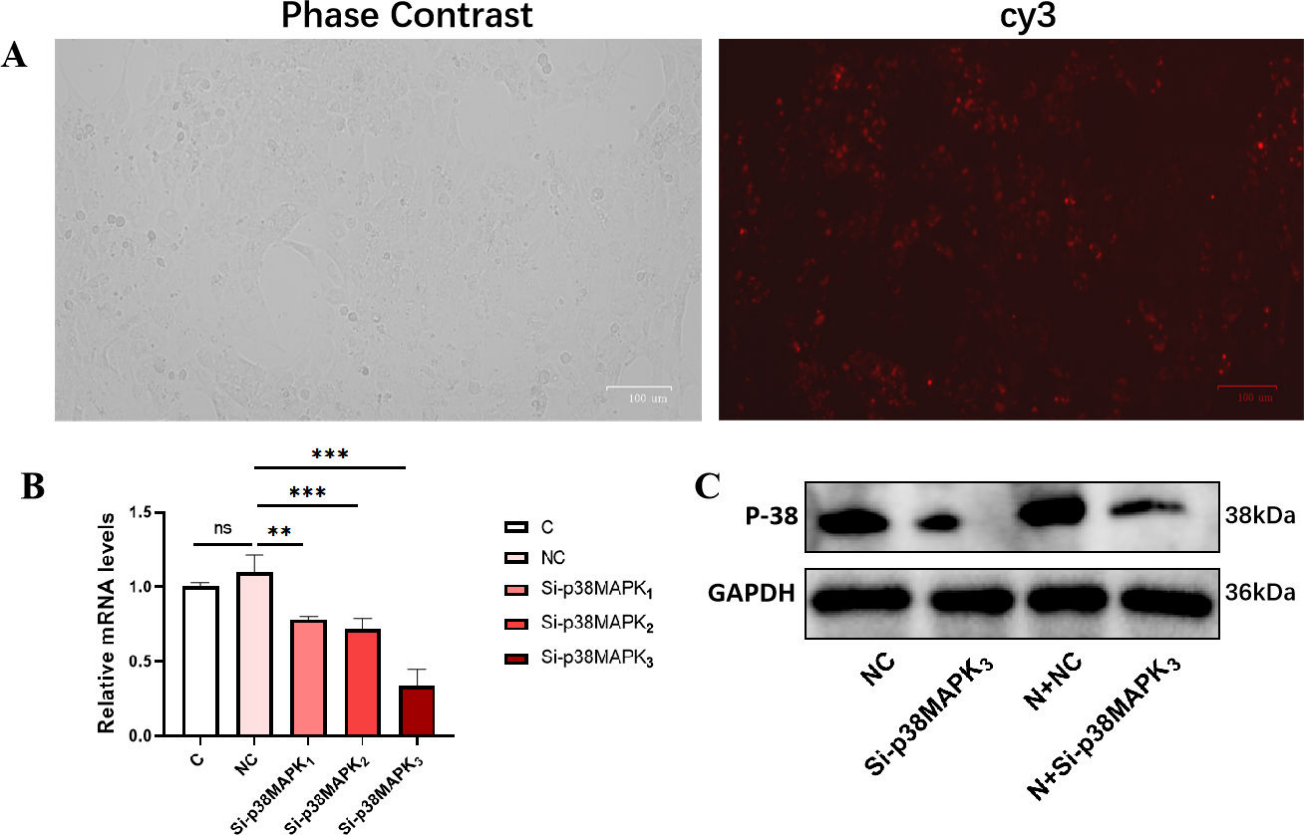
Results related to intervention with knockdown. (**A**) cy3 fluorescent labeling (red). (**B**) Knockdown efficiency of p38 MAPK mRNA levels (*n* = 4). (**C**) Knockdown efficiency of p38 MAPK protein expression level. The values were expressed in the format mean ± SD (*n* = 3). ***P < 0.01, ***P < 0.001 compared with C or NC group. C, control group; N, NIBV group; NC, empty vector control group.

**Fig 10:**
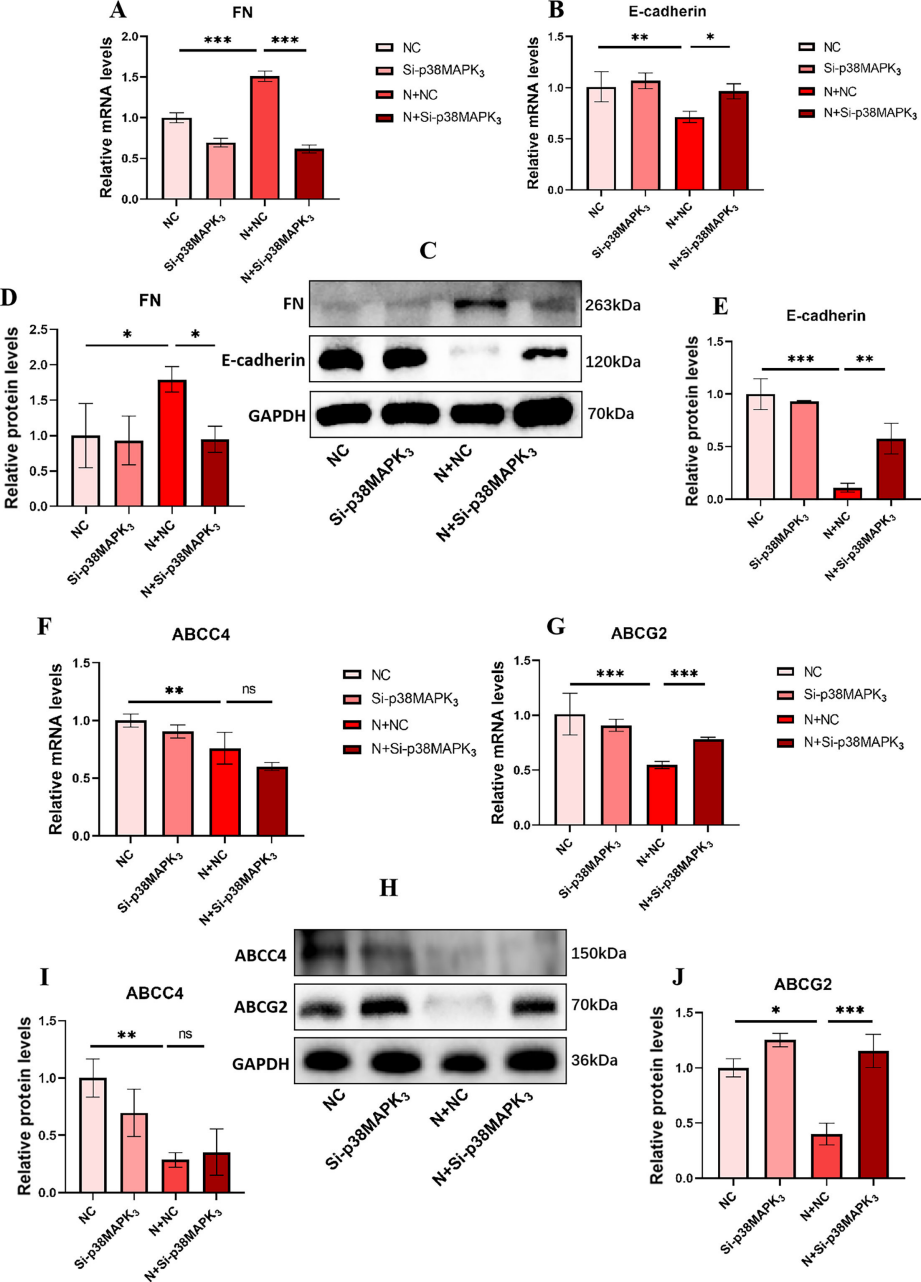
(**A, B**) qPCR detection of gene expression results of EMT-related proteins (*n* = 4). (**C**) The expression of EMT excretion-related proteins after p38 MAPK knockdown. Grayscale quantitative analysis of protein bands: FN (**D**) and E-cadherin (**E, F, G**) qPCR detection of gene expression results of uric acid excretion-related protein (*n* = 4). (**H**) The expression of uric acid excretion-related proteins after p38 MAPK knockdown. Grayscale quantitative analysis of protein bands: ABCC4 (**I**) and ABCG2 (**J**). The values were expressed in the format mean ± SD (*n* = 3). *P < 0.05, **P < 0.01, ***P < 0.001 compared with NC or NC + NIBV group. C, control group; N, NIBV group; NC, empty vector control group.

To confirm that the occurrence of EMT is induced via the p38 MAPK pathway, we knocked it down using siRNA. Cy3 fluorescence labeling revealed that the plasmids were successfully transfected into the cells (Fig. 9A). We subsequently assessed the knockout efficiency of p38 MAPK (Fig. 9B and C). In addition, compared with that in the Con group, the expression of E-cadherin in the NIBV group significantly decreased, while the expression of FN significantly increased, suggesting that EMT occurred in renal tubular epithelial cells. Compared with that in the NIBV group, the expression of mesenchymal transition proteins in the p38 MAPK knockdown group was reversed, indicating that blocking the p38 MAPK pathway could prevent NIBV-induced EMT in renal tubular epithelial cells (Fig. 10A through E). The expression of uric acid transport-related proteins was subsequently detected in the four groups. Compared with that in the NIBV group, the expression of the uric acid excretion protein ABCG2 in the Si-p38 MAPK group was significantly increased, while the expression of ABCC4 was not reversed, which also verified that ABCG2 was the main contributor to uric acid excretion in the late stage of NIBV infection (Fig. 10F through J). The siRNA knockdown results were consistent with the outcomes of p38 MAPK inhibitor (SB203580) treatment (Fig. S2 and S3). In summary, the results of the siRNA assay indicated that NIBV induced EMT by activating the p38 MAPK pathway, thereby inhibiting the expression of ABCG2.

## DISCUSSION

In poultry infected with NIBV, there is often an abnormal surge in uric acid levels within their bodies, resulting in hyperuricemia. These conditions subsequently cause loose white stools, slowed growth, and even sudden death, causing losses to the poultry breeding industry (35, 36). The primary target organ of NIBV infection is the kidney, and the kidney serves as the main pathway through which poultry excrete uric acid (10, 30). Hence, we hypothesized that the damage inflicted on the kidney by NIBV inevitably affects the excretion of uric acid. Therefore, our research focuses on the influence of NIBV on uric acid excretion.

The kidneys regulate the homeostatic balance of uric acid in the body by relying on the coordinated operation of uric acid transporters located in renal tubular epithelial cells (11). Uric acid is excreted from the kidneys through reabsorption and subsequent excretion. Research by Minghui Wang et al. indicated that ABCG2, ABCC4, and Slc2a9 play crucial roles in uric acid transport in chickens (37–39). The peak value at 11 dpi in the viral load measurement results is in line with the changing trend of serum uric acid levels in chicks, which is also in accordance with the results presented by previous studies (31, 40). The expression of uric acid reabsorption proteins does not increase but rather decreases when uric acid levels are at their highest, revealing that the cause of elevated uric acid is not uric acid reabsorption proteins. This leads us to focus our subsequent research on uric acid excretion proteins. Prestin K et al. reported that transient HNF4α overexpression enhanced the activity of the hepatocyte nuclear factor (HNF) 4α binding site in the Slc2a9 isoform 1 promoter, whereas mutations in the binding site reduced activation (41). These findings indicate that Slc2a9 expression is indeed reduced under certain circumstances. The mechanism by which NIBV causes a reduction in Slc2a9 expression has not been explored in depth here because our focus is not on reabsorption proteins. We will further explore this topic if necessary.

Interestingly, in the later stages of NIBV modeling, the expression of the uric acid excretion-related protein ABCG2 was restored and even significantly increased, indicating that the ability of the kidney to excrete uric acid at this time depended mainly on ABCG2. Previous studies on chickens revealed that renal urate transport is compromised in cases of decreased renal function and that extrarenal ABCG2 seems to play a compensatory role (42), and our results align with theirs, suggesting that ABCG2 compensates for reduced ABCC4 expression. The increased compensatory expression is sufficient to restore chicks with abnormally elevated uric acid levels back to normal. However, an intriguing question is why the trends of ABCG2 and ABCC4 in the later stages of NIBV modeling are completely opposite. The expression and localization of both ABCG2 and ABCC4 within chicken kidneys are particularly critical for understanding this phenomenon. Notably, proteins typically located at the apical membrane (luminal side) of the renal tubules serve as excretory proteins that are responsible for transporting substances from renal tubular cells into the tubular lumen. Conversely, transporters located at the basolateral membrane function as reabsorptive proteins, tasked with reclaiming certain substances from the urine back into the circulation (32, 33). Our double immunofluorescence staining results indicate that the expression and localization of ABCG2 and ABCC4 are not entirely identical. Additionally, studies by Yuting Wu et al. have shown that impaired uric acid excretion in chickens is associated with ABCG2 (BCRP) (43). We hypothesize that, in the late stage of NIBV infection, ABCG2 may serve as the primary transporter involved in uric acid excretion. Based on this premise, we will guide our subsequent research efforts to focus on ABCG2.

Previous studies have indicated that cytokines and growth factors can significantly influence the expression of the ABCG2 gene (22, 44). Yin et al. reported a reduction in ABCG2 gene expression when the ABCG2-positive cell population was isolated from MCF-7 cells and subjected to TGF-β treatment. The decrease in ABCG2 expression is strongly suppressed during TGF-β-directed EMT and is restored when cells return to an epithelial phenotype (22). The trends observed in ABCG2 expression were consistent with these findings. Consequently, we subsequently focused on exploring the relationship between EMT and ABCG2 expression. The *in vivo* results revealed that the expression of ABCG2 decreased at the time when EMT occurred, and in the later stages of NIBV modeling, ABCG2 expression was also restored when the epithelial phenotype was restored. This reversible repair may benefit from the reversibility of the EMT. Research has consistently demonstrated that the reverse process of EMT, referred to as MET, occurs frequently during developmental processes such as heart development, kidney morphogenesis, and somite formation, as well as in cancer (45, 46).

Studies have shown that p38-MAPK is one of the pathways that mediates cellular EMT. p38 can be phosphorylated by TGF-β to form active p-p38 (23, 25). We previously reported that the suppression of ABCG2 expression is influenced by the EMT induced by TGF-β. Thus, we believe that p38 MAPK is the “connector” between TGF-β and NIBV-induced EMT. To investigate this hypothesis, we detected the gene and protein levels of the p38 MAPK pathway at various time points *in vivo* and *in vitro*. The p38 MAPK pathway is indeed activated during EMT. Furthermore, in the stage of epithelial phenotype recovery following NIBV *in vivo* modeling, the levels of phosphorylated p38 (p-p38) were comparable to those observed in the normal group. Furthermore, to further validate that NIBV-induced EMT in renal tubular epithelial cells is mediated by p38 MAPK, we knocked down p38 MAPK expression using small interfering RNA. After p38 MAPK knockdown, NIBV failed to induce EMT in renal tubular epithelial cells, and the expression of ABCG2 was minimally affected. These findings indicate that NIBV-induced EMT in renal tubular epithelial cells is mediated through the p38-MAPK signaling pathway.

Our research provides a foundation for the potential of interventions to regulate uric acid levels in poultry, with the goal of preventing and alleviating chicken gout associated with NIBV infection. Additionally, this study provides valuable insights for the development of therapeutic drugs targeting hyperuricemia and avian gout in chickens. Uric acid metabolism in poultry and mammals is not identical, and a homology analysis of the ABCG2 gene also revealed significant differences between avian species and humans (Fig. S4). Our research contributes to the understanding of uric acid metabolism mechanisms in poultry.

### Conclusion

NIBV infection typically causes an abnormal elevation in uric acid levels in chickens. The mechanism by which NIBV causes an abnormal increase in uric acid levels in chickens involves the induction of renal tubular epithelial cell EMT through the TGF-β/P-P38 pathway and the subsequent strong inhibition of ABCG2 expression, causing uric acid excretion disorders in chickens. This study elucidates the mechanism through which NIBV induces an increase in uric acid levels in chicks, laying the foundation for the prevention and alleviation of NIBV infection-induced gout in chickens.

## Data Availability

All relevant data are within the article.

## SUPPLEMENTAL MATERIAL

Supplemental figures jvi.01031-25-s0001.docx Figures S1 to S4.

Graphical abstract jvi.01031-25-s0002.docx Visual depiction of the study.

ASM does not own the copyrights to Supplemental Material that may be linked to, or accessed through, an article. The authors have granted ASM a non-exclusive, world-wide license to publish the Supplemental Material files. Please contact the corresponding author directly for reuse.

## References

[1] Whitton C, Bogueva D, Marinova D, Phillips CJC. 2021. Are we approaching peak meat consumption? Analysis of meat consumption from 2000 to 2019 in 35 countries and its relationship to gross domestic product. Animals (Basel) 11:3466. doi:10.3390/ani11123466 34944243 PMC8697883

[2] Yin HC, Liu ZD, Zhang WW, Yang QZ, Yu TF, Jiang XJ. 2022. Chicken intestinal microbiota modulation of resistance to nephropathogenic infectious bronchitis virus infection through IFN-I. Microbiome 10:162. doi:10.1186/s40168-022-01348-2 36192807 PMC9527382

[3] Chen H, Shi W, Feng S, Yuan L, Jin M, Liang S, Wang X, Si H, Li G, Ou C. 2024. A novel highly virulent nephropathogenic QX-like infectious bronchitis virus originating from recombination of GI-13 and GI-19 genotype strains in China. Poult Sci 103:103881. doi:10.1016/j.psj.2024.103881 38865766 PMC11223121

[4] Sharma M, Rahman FAT, Sharma G, Dey S, Chellappa MM, Sharma A, Dhama K, Saikumar G, Kumar AM. 2024. Immuno-pathogenesis study of local infectious bronchitis virus G1-1 lineage variant showed altered tissue tropism in experimental broiler chickens. Vet Res Commun 48:3683–3697. doi:10.1007/s11259-024-10525-7 39222200

[5] Matthijs MGR, van Eck JHH, Landman WJM, Stegeman JA. 2003. Ability of Massachusetts-type infectious bronchitis virus to increase colibacillosis susceptibility incommercial broilers: a comparison between vaccine and virulent field virus. Avian Pathol 32:473–481. doi:10.1080/0307945031000154062 14522702

[6] Zhang Y, Xu Z, Cao Y. 2021. Host antiviral responses against avian infectious bronchitis virus (IBV): focus on innate immunity. Viruses 13:1698. doi:10.3390/v13091698 34578280 PMC8473314

[7] Bande F, Arshad SS, Omar AR, Hair-Bejo M, Mahmuda A, Nair V. 2017. Global distributions and strain diversity of avian infectious bronchitis virus: a review. Anim Health Res Rev 18:70–83. doi:10.1017/S1466252317000044 28776490

[8] Afanador G, Roberts JR. 1994. Effect of nephropathogenic infectious bronchitis viruses on renal function in young male broiler chickens. Br Poult Sci 35:445–456. doi:10.1080/00071669408417709 7953788

[9] Mandal AK, Mount DB. 2015. The molecular physiology of uric acid homeostasis. Annu Rev Physiol 77:323–345. doi:10.1146/annurev-physiol-021113-170343 25422986

[10] Wu W, Xu R, Lv Y, Bao E. 2020. Goose astrovirus infection affects uric acid production and excretion in goslings. Poult Sci 99:1967–1974. doi:10.1016/j.psj.2019.11.064 32241477 PMC7587898

[11] Dalbeth N, Gosling AL, Gaffo A, Abhishek A. 2021. Gout. Lancet 397:1843–1855. doi:10.1016/S0140-6736(21)00569-9 33798500

[12] Adomako EA, Moe OW. 2023. Uric acid transport, transporters, and their pharmacological targeting. Acta Physiol (Oxf) 238:e13980. doi:10.1111/apha.13980 37092855

[13] Du L, Zong Y, Li H, Wang Q, Xie L, Yang B, Pang Y, Zhang C, Zhong Z, Gao J. 2024. Hyperuricemia and its related diseases: mechanisms and advances in therapy. Signal Transduct Target Ther 9:212. doi:10.1038/s41392-024-01916-y 39191722 PMC11350024

[14] Eckenstaler R, Benndorf RA. 2021. The role of ABCG2 in the pathogenesis of primary hyperuricemia and gout-an update. Int J Mol Sci 22:6678. doi:10.3390/ijms22136678 34206432 PMC8268734

[15] Zhang YC, Zhang YT, Wang Y, Zhao Y, He LJ. 2023. What role does PDL1 play in EMT changes in tumors and fibrosis? Front Immunol 14:1226038. doi:10.3389/fimmu.2023.1226038 37649487 PMC10463740

[16] Nieto MA, Huang RY-J, Jackson RA, Thiery JP. 2016. EMT: 2016. Cell 166:21–45. doi:10.1016/j.cell.2016.06.028 27368099

[17] Serrano-Gomez SJ, Maziveyi M, Alahari SK. 2016. Regulation of epithelial-mesenchymal transition through epigenetic and post-translational modifications. Mol Cancer 15:18. doi:10.1186/s12943-016-0502-x 26905733 PMC4765192

[18] Nowak E, Bednarek I. 2021. Aspects of the epigenetic regulation of EMT related to cancer metastasis. Cells 10:3435. doi:10.3390/cells10123435 34943943 PMC8700111

[19] David CJ, Massagué J. 2018. Contextual determinants of TGFβ action in development, immunity and cancer. Nat Rev Mol Cell Biol 19:419–435. doi:10.1038/s41580-018-0007-0 29643418 PMC7457231

[20] Pickup M, Novitskiy S, Moses HL. 2013. The roles of TGFβ in the tumour microenvironment. Nat Rev Cancer 13:788–799. doi:10.1038/nrc3603 24132110 PMC4025940

[21] Lee JH, Massagué J. 2022. TGF-β in developmental and fibrogenic EMTs. Semin Cancer Biol 86:136–145. doi:10.1016/j.semcancer.2022.09.004 36183999 PMC10155902

[22] Yin L, Castagnino P, Assoian RK. 2008. ABCG2 expression and side population abundance regulated by a transforming growth factor beta-directed epithelial-mesenchymal transition. Cancer Res 68:800–807. doi:10.1158/0008-5472.CAN-07-2545 18245481

[23] Munkonda MN, Akbari S, Landry C, Sun S, Xiao F, Turner M, Holterman CE, Nasrallah R, Hébert RL, Kennedy CRJ, Burger D. 2018. Podocyte-derived microparticles promote proximal tubule fibrotic signaling via p38 MAPK and CD36. J Extracell Vesicles 7:1432206. doi:10.1080/20013078.2018.1432206 29435202 PMC5804677

[24] Avila-Carrasco L, Majano P, Sánchez-Toméro JA, Selgas R, López-Cabrera M, Aguilera A, González Mateo G. 2019. Natural plants compounds as modulators of epithelial-to-mesenchymal transition. Front Pharmacol 10:715. doi:10.3389/fphar.2019.00715 31417401 PMC6682706

[25] Hickson JA, Huo D, Vander Griend DJ, Lin A, Rinker-Schaeffer CW, Yamada SD. 2006. The p38 kinases MKK4 and MKK6 suppress metastatic colonization in human ovarian carcinoma. Cancer Res 66:2264–2270. doi:10.1158/0008-5472.CAN-05-3676 16489030

[26] Majumder J, Minko T. 2021. Targeted nanotherapeutics for respiratory diseases: cancer, fibrosis, and coronavirus. Adv Ther (Weinh) 4:2000203. doi:10.1002/adtp.202000203 33173809 PMC7646027

[27] Uhler C, Shivashankar GV. 2021. Mechanogenomic coupling of lung tissue stiffness, EMT and coronavirus pathogenicity. Curr Opin Solid State Mater Sci 25:100874. doi:10.1016/j.cossms.2020.100874 33519291 PMC7833345

[28] George PM, Wells AU, Jenkins RG. 2020. Pulmonary fibrosis and COVID-19: the potential role for antifibrotic therapy. Lancet Respir Med 8:807–815. doi:10.1016/S2213-2600(20)30225-3 32422178 PMC7228727

[29] Niayesh-Mehr R, Kalantar M, Bontempi G, Montaldo C, Ebrahimi S, Allameh A, Babaei G, Seif F, Strippoli R. 2024. The role of epithelial-mesenchymal transition in pulmonary fibrosis: lessons from idiopathic pulmonary fibrosis and COVID-19. Cell Commun Signal 22:542. doi:10.1186/s12964-024-01925-y 39538298 PMC11558984

[30] Qi Q, Li Y, Ding M, Huang C, Omar SM, Shi Y, Liu P, Cai G, Zheng Z, Guo X, Gao X. 2024. Wogonin inhibits apoptosis and necroptosis induced by nephropathogenic infectious bronchitis virus in chicken renal tubular epithelial cells. Int J Mol Sci 25:8194. doi:10.3390/ijms25158194 39125764 PMC11312162

[31] Chen W, Huang C, Shi Y, Li N, Wang E, Hu R, Li G, Yang F, Zhuang Y, Liu P, Hu G, Gao X, Guo X. 2022. Investigation of the crosstalk between GRP78/PERK/ATF-4 signaling pathway and renal apoptosis induced by nephropathogenic infectious bronchitis virus infection. J Virol 96:e0142921. doi:10.1128/JVI.01429-21 34669445 PMC8791289

[32] Park JH, Jo YI, Lee JH. 2020. Renal effects of uric acid: hyperuricemia and hypouricemia. Korean J Intern Med 35:1291–1304. doi:10.3904/kjim.2020.410 32872730 PMC7652664

[33] Wen S, Arakawa H, Tamai I. 2024. Uric acid in health and disease: from physiological functions to pathogenic mechanisms. Pharmacol Ther 256:108615. doi:10.1016/j.pharmthera.2024.108615 38382882

[34] Li Y, Qi Q, Chen Y, Ding M, Huang M, Huang C, Liu P, Gao X, Guo X, Zheng Z. 2025. RIPK3 activation of CaMKII triggers mitochondrial apoptosis in NIBV-infected renal tubular epithelial cells. Vet Microbiol 302:110375. doi:10.1016/j.vetmic.2025.110375 39808936

[35] Li N, Huang C, Chen W, Li Z, Hu G, Li G, Liu P, Hu R, Zhuang Y, Luo J, Gao X, Guo X. 2022. Nephropathogenic infectious bronchitis virus mediates kidney injury in chickens via the TLR7/NF-κB signaling axis. Front Cell Infect Microbiol 12:865283. doi:10.3389/fcimb.2022.865283 35402297 PMC8983847

[36] Chen Y, Feng C, Huang C, Shi Y, Omar SM, Zhang B, Cai G, Liu P, Guo X, Gao X. 2024. Preparation of polyclonal antibodies to chicken P62 protein and its application in nephropathogenic infectious bronchitis virus-infected chickens. Int J Biol Macromol 271:132515. doi:10.1016/j.ijbiomac.2024.132515 38768912

[37] Wang M, Wu J, Jiao H, Oluwabiyi C, Li H, Zhao J, Zhou Y, Wang X, Lin H. 2022. Enterocyte synthesizes and secrets uric acid as antioxidant to protect against oxidative stress via the involvement of Nrf pathway. Free Radic Biol Med 179:95–108. doi:10.1016/j.freeradbiomed.2021.12.307 34954337

[38] Jiang Z, Cao J, Su H, Cao H, Sun Z, Jiang H, Fan Y. 2022. Exercise serum regulates uric acid transporters in normal rat kidney cells. Sci Rep 12:18086. doi:10.1038/s41598-022-22570-w 36302802 PMC9613886

[39] Nakanishi T, Ohya K, Shimada S, Anzai N, Tamai I. 2013. Functional cooperation of URAT1 (SLC22A12) and URATv1 (SLC2A9) in renal reabsorption of urate. Nephrol Dial Transplant 28:603–611. doi:10.1093/ndt/gfs574 23291366

[40] Xu P, Shi Y, Liu P, Yang Y, Zhou C, Li G, Luo J, Zhang C, Cao H, Hu G, Guo X. 2020. 16S rRNA gene sequencing reveals an altered composition of the gut microbiota in chickens infected with a nephropathogenic infectious bronchitis virus. Sci Rep 10:3556. doi:10.1038/s41598-020-60564-8 32103130 PMC7044311

[41] Prestin K, Wolf S, Feldtmann R, Hussner J, Geissler I, Rimmbach C, Kroemer HK, Zimmermann U, Meyer zu Schwabedissen HE. 2014. Transcriptional regulation of urate transportosome member SLC2A9 by nuclear receptor HNF4α. Am J Physiol Renal Physiol 307:F1041–51. doi:10.1152/ajprenal.00640.2013 25209865

[42] Ding X, Li M, Peng C, Wang Z, Qian S, Ma Y, Fang T, Feng S, Li Y, Wang X, Li J, Wu J. 2019. Uric acid transporters BCRP and MRP4 involved in chickens uric acid excretion. BMC Vet Res 15:180. doi:10.1186/s12917-019-1886-9 31146764 PMC6543625

[43] Wu Y, Liu J, Liu S, Fan W, Ding C, Gao Z, Tang Z, Luo Y, Shi X, Tan L, Song S. 2022. Bromoacetic acid causes oxidative stress and uric acid metabolism dysfunction via disturbing mitochondrial function and Nrf2 pathway in chicken kidney. Environ Toxicol 37:2910–2923. doi:10.1002/tox.23647 36017758

[44] Robey RW, To KKK, Polgar O, Dohse M, Fetsch P, Dean M, Bates SE. 2009. ABCG2: a perspective. Adv Drug Deliv Rev 61:3–13. doi:10.1016/j.addr.2008.11.003 19135109 PMC3105088

[45] Polyak K, Weinberg RA. 2009. Transitions between epithelial and mesenchymal states: acquisition of malignant and stem cell traits. Nat Rev Cancer 9:265–273. doi:10.1038/nrc2620 19262571

[46] Huang Y, Hong W, Wei X. 2022. The molecular mechanisms and therapeutic strategies of EMT in tumor progression and metastasis. J Hematol Oncol 15:129. doi:10.1186/s13045-022-01347-8 36076302 PMC9461252

