# Supplementary material for: Nephropathogenic infectious bronchitis virus induces epithelial-mesenchymal transition of renal tubular epithelial cells through the TGF-β/p-P38 pathway causing uric acid excretion disorder in chickens
Source: J Virol. 2025 Oct 14;99(11):e01031-25. doi: 10.1128/jvi.01031-25 (PMC12645968; doi:10.1128/jvi.01031-25)
Supplement: Supplemental figures — Figures S1 to S4. [file jvi.01031-25-s0001.docx]

Supplementary materials


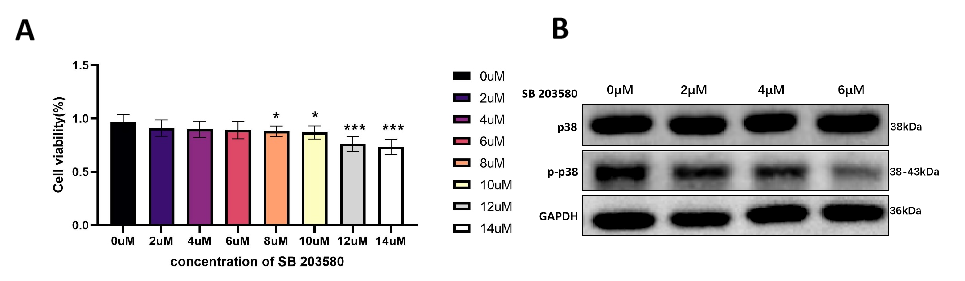


**Fig S1.** A. CCK8 detects cell viability when cells are incubated with different inhibitor concentrations for 24 hours. (n=8) B. The inhibitory effects at various concentrations.


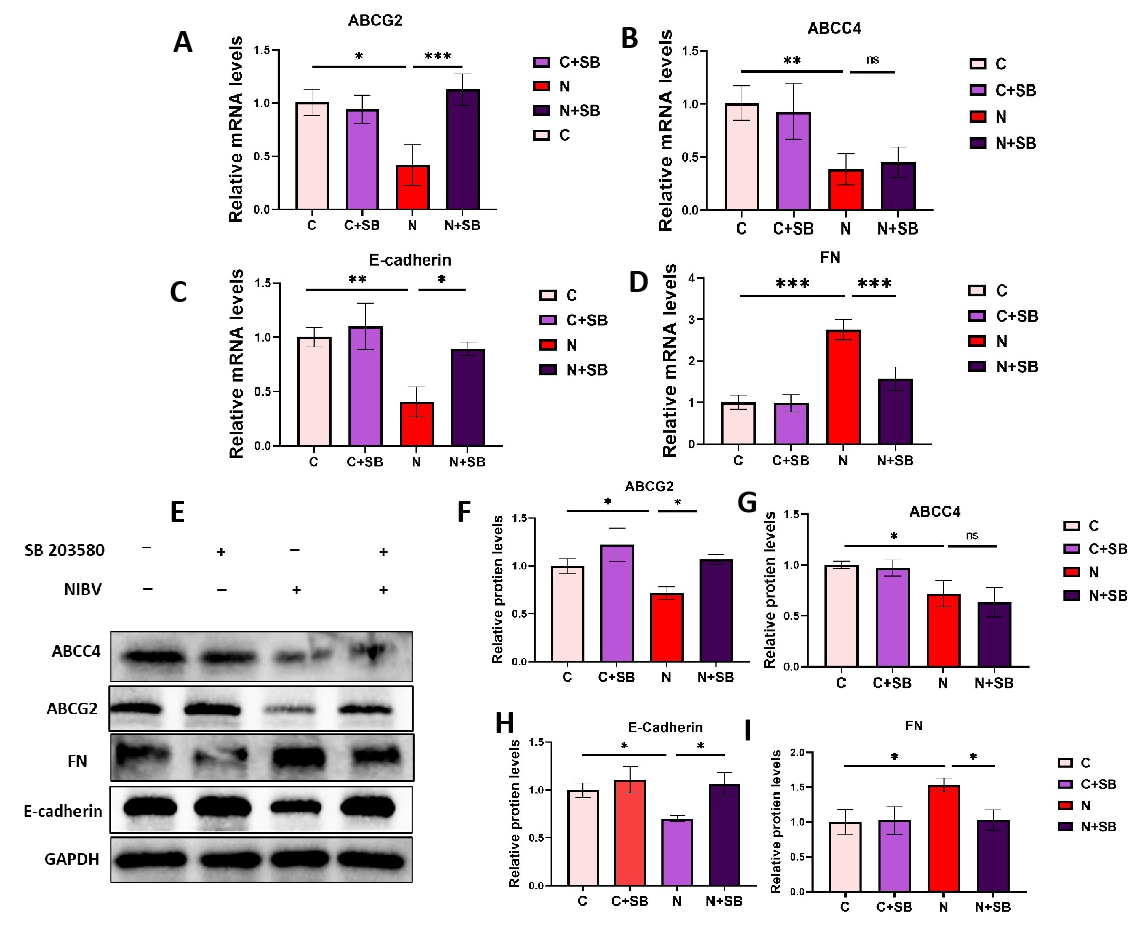


**Fig S2.** Results related to intervention with inhibitor SB203580. A. qPCR detection of gene expression results of uric acid excretion proteins ABCG2(C) and ABCC4(D). (n=4) E.F. qPCR detection of gene expression results of EMT-related proteins. (n=4) G. Protein bands of EMT and uric acid excretion-related proteins after inhibitor intervention. Gray scale quantitative analysis of protein bands, ABCC4(H), ABCG2(I), E-cadherin(J), FN(K) The values were expressed in the format mean ± SD.(n=3) *P<0.05, **P < 0.01, ***P < 0.001 compared with CON or NIBV group.

**
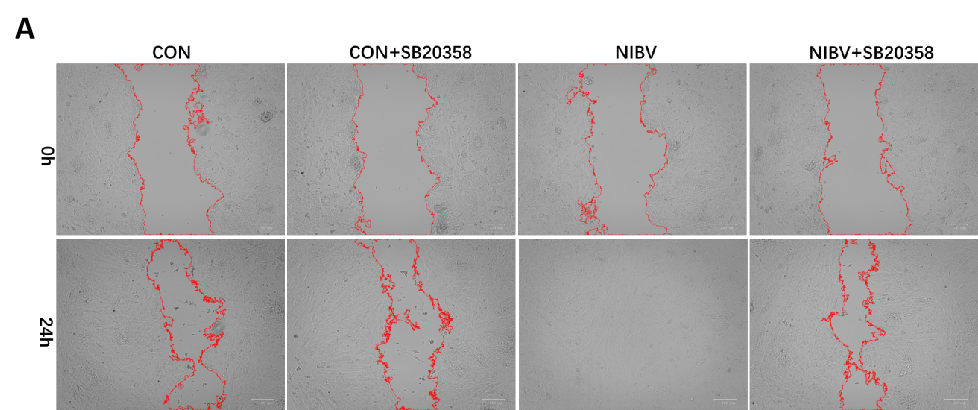
**

**Fig S3**. A. Scratch experiment. The cell-free area is between the two red marking lines, and the marked area is identified and marked by Image J.


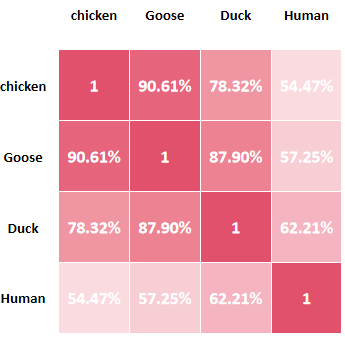


**Fig S4**. A. Homology comparison of ABCG2 mRNA sequences among chickens, geese, ducks, and humans. Download sequences from NCBI and perform alignment in DNAMAN software.
