# Supplementary figures and images for: Nephropathogenic infectious bronchitis virus induces epithelial-mesenchymal transition of renal tubular epithelial cells through the TGF-β/p-P38 pathway causing uric acid excretion disorder in chickens
Source: J Virol. 2025 Oct 14;99(11):e01031-25. doi: 10.1128/jvi.01031-25 (PMC12645968; doi:10.1128/jvi.01031-25)

**Graphical abstract**


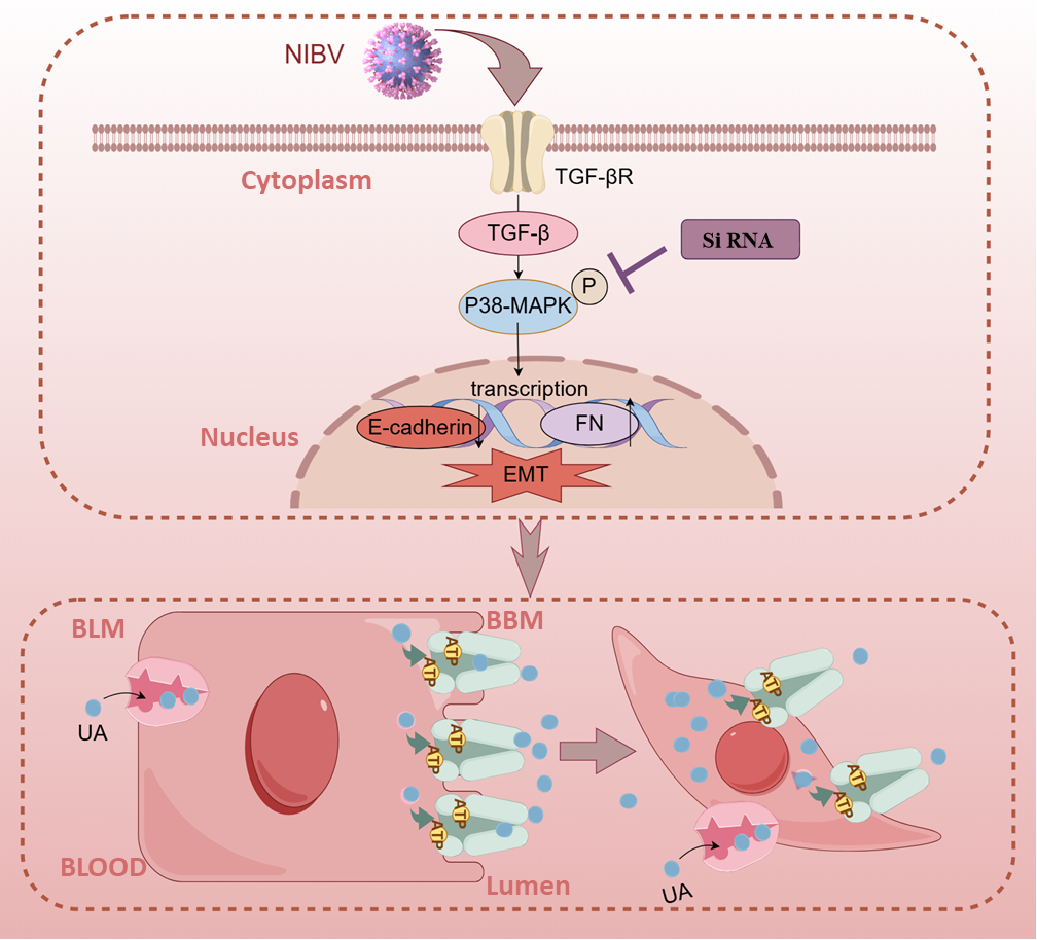


Drawn in Fig Draw, export ID: WSPWTcceb8.

Supplement: Graphical abstract — Visual depiction of the study. [file jvi.01031-25-s0002.docx]
